# Austerity policies and falling life expectancy in disadvantaged areas in Scotland: a pre-pandemic decomposition analysis

**DOI:** 10.1093/eurpub/ckag076

**Published:** 2026-05-15

**Authors:** Maria Kaye-Bardgett, Julie Ramsay, Gerry McCartney, Grant Wyper, David Walsh

**Affiliations:** National Records of Scotland, Edinburgh, United Kingdom; National Records of Scotland, Edinburgh, United Kingdom; School of Social and Political Sciences, University of Glasgow, Glasgow, United Kingdom; School of Health & Wellbeing, University of Glasgow, Glasgow, United Kingdom; School of Health & Wellbeing, University of Glasgow, Glasgow, United Kingdom

## Abstract

Previous decomposition analyses quantified the contribution of changes in age- and cause-specific mortality to the adverse changes in life expectancy observed in the UK since 2012. We extend those analyses by stratifying by socioeconomic deprivation. Between 2012–14 and 2017–19, changes in life expectancy were substantially worse in Scotland’s 20% most deprived areas. Negative impacts on life expectancy were found for most age groups and causes of death, reversing improvements between 2001–03 and 2012–14. This is consistent with the evidence of the role of UK Government ‘austerity’ policies in reversing previous health improvements among the UK’s poorest populations.

## Introduction

*Note: additional supporting references for this paper are included in the online appendix.*

Profound changes to life expectancy have been observed across the UK since the early 2010s. In all four nations (England, Scotland, Northern Ireland, and Wales), long-term national-level improvements effectively ground to a halt, while in the poorest areas, life expectancy actually declined. These changes predate the COVID-19 pandemic and post-pandemic period of high inflation (‘cost of living crisis’) but have been made worse by both. A large body of evidence has demonstrated that UK Government ‘austerity’ policies, initiated in 2010 but still largely in place today, are the most likely cause of these changes [1]. In the UK context, austerity can be crudely defined as large-scale cuts in government spending, and evidence shows that a combination of cuts to the provision of vital public services and historically unparalleled reductions in social security provision have had profound effects on the health and mortality profile of the poorest in society. The causal pathways involved, relating to poverty and other key social determinants of health, are well understood and have been set out in detail elsewhere [1].

Key to our understanding of these effects was a set of decomposition analyses, published in 2020, which showed that the national-level stalling of improvement in life expectancy was driven by adverse changes in mortality—i.e. increased rates or slower improvement in rates—for almost *all* age groups and *all* major causes of death [2]. This was important in demonstrating that the overall changes to life expectancy were neither explained by changes in rates of specific, individual, causes of death nor by changes for particular age groups, as had been previously hypothesized; rather, the analyses showed that a common underlying factor affecting multiple, diverse, causes of death across different age groups was a more likely explanation [1]. These decomposition analyses were undertaken with Scottish mortality data; however, similar results were also shown elsewhere in the UK [3]. One weakness of the Scottish analyses was that they were not stratified by socioeconomic deprivation. This is an important limitation, given that we know that the changes to life expectancy have been demonstrably worse in poorer areas. The aim here, therefore, was to extend the analyses by comparing the contribution of age- and cause-specific mortality changes to life expectancy trends between the least and most deprived areas of Scotland to establish whether, and to what extent, those contributions were more pronounced in more disadvantaged areas.

## Methods

We replicated the previously employed methodology [2], additionally stratifying by the least and most deprived 20% of the Scottish population using the Scottish Index of Multiple Deprivation (SIMD), a neighbourhood-level (c. 750 population) measure of relative socioeconomic deprivation, calculated from administrative data, and used in official government publications and resource allocation [4]. Different versions of SIMD were used for different time periods (Supplementary Table S1). We calculated period life expectancy (LE) at birth from National Records of Scotland mortality and population data, and then applied Arriaga’s method of life expectancy decomposition [5], thereby estimating the contribution of different underlying causes of death, and different age groups, to changes in life expectancy over time, and between the most and least deprived 20% of Scottish neighbourhoods.

LE calculations were based on 5-year age groups, with the exceptions of the 0-4 (split into 0 and 1-4 years) and 90+ groups. For robustness, 3 years of data (e.g. 2001–03) were combined. Twenty-six major causes of death were used (25 for females, that is excluding prostate cancer), defined by the previously used ICD10 codes (Supplementary Table S2). The results are shown as the annualized change in LE in weeks per year.

Aside from stratification by deprivation, the only other difference to the original methodology was that we extended the period of analysis from 2017 to 2019. This updates the original analyses slightly but avoids the additional complication of factoring in the effects of the COVID-19 pandemic. Thus, we compared changes in LE in the period 2001–03 to 2012–14 with those in 2012–14 to 2017–19. The break between periods was based on previous estimates of when the LE trends in Scotland changed [2].

## Results

Overall, for females living in the most deprived 20% of areas in Scotland, the annualized increase in LE between 2001–03 and 2012–14 was 8.5 weeks per year. However, in the second period, that was reversed: LE fell by 8.6 weeks/year. In contrast, for those in the least deprived areas, LE increased in both periods: by 10.4 weeks/year in the first, and by 8.4 weeks/year in the second (Supplementary Table S3).

The overall results for males were similar (also Supplementary Table S2). In the most deprived areas, a 17.9 weeks/year increase in the first period was followed by an 8.6 weeks/year *decrease* in the second period. In contrast, in the least deprived areas, LE increased in both periods, although to a lesser extent in 2012–2014 (falling from 15.2 to 5.8 weeks/year).

**Figure 1.**
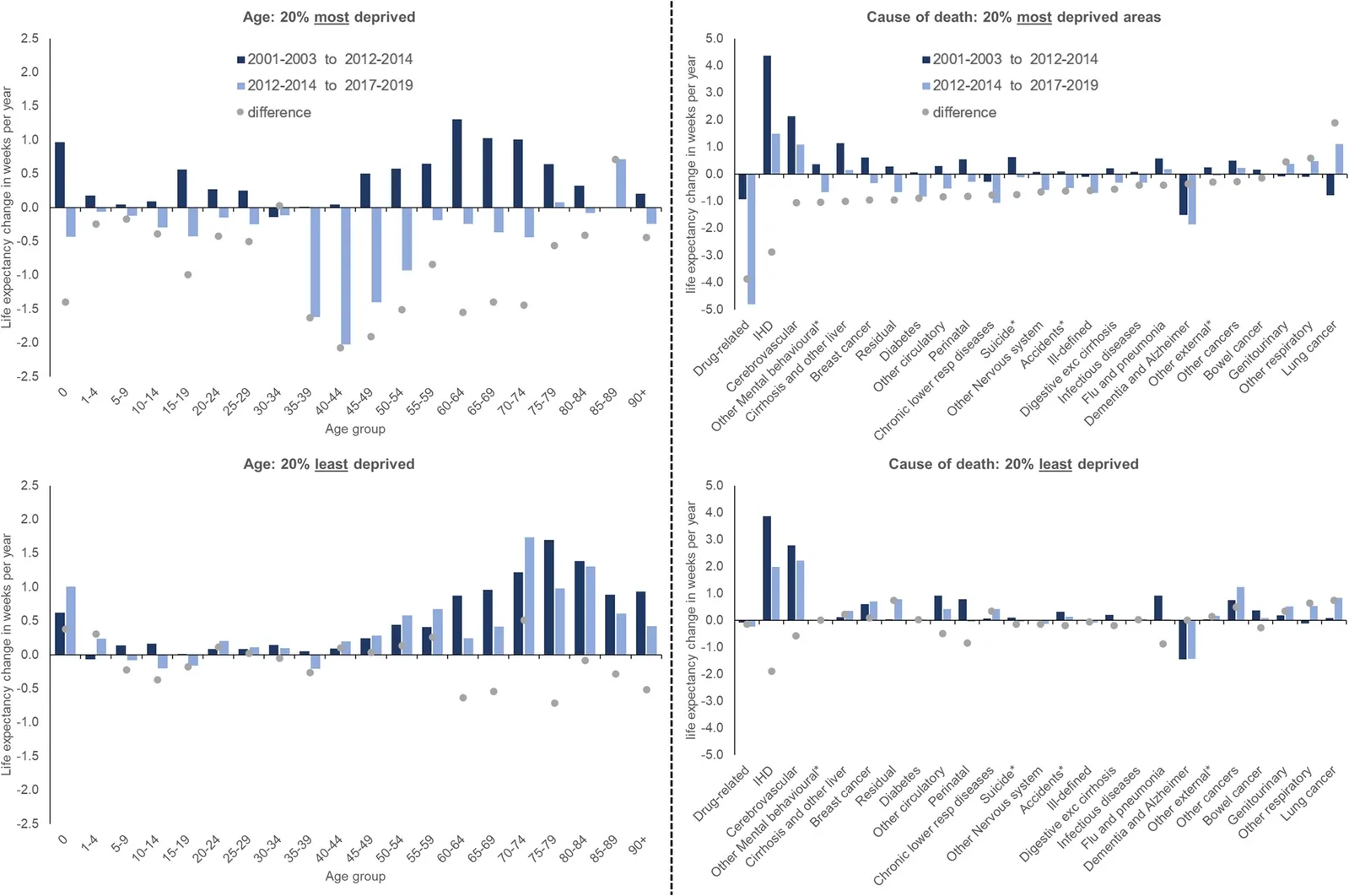
Decomposition of the contribution of age at death (left-hand charts), and specific cause of death (right-hand charts), to changes in female life expectancy between 2001–03 and 2012–14, and 2012–14 and 2017–19, for the populations living in the 20% most deprived (top charts) and 20% least deprived (bottom charts) areas. *Excluding causes that are included under drug-related deaths. IHD, ischaemic heart disease.

Figure 1 presents the main decomposition results for females; Supplementary Fig. S1 in shows the results for males.

The figure compares the contribution of changes in mortality to changes in LE between the two periods for each age group (left-hand charts) and for each cause of death (right-hand charts). The top charts are for the 20% most deprived areas in Scotland; the bottom charts are for the 20% least deprived areas. The dark and light bars show the annualized change in LE in the first and second periods, respectively; the circles represent the difference between the two periods. Bars above the x-axis represent positive change (i.e. an improvement in LE); bars below represent the opposite.

First, there are clear differences in age-specific patterns between the most and least deprived areas. For those living in the most deprived areas, reductions—rather than just slower improvements—in LE can be seen for all except two age groups (one of which is a negligible increase). In contrast, in the least deprived areas, only four age groups saw reductions (and only marginally). Furthermore, for nine age groups in the least deprived areas, LE improved to a greater degree in the second period than in the first; in the most deprived areas, that was only the case for one age group (85–89 years). In the most deprived areas, the largest negative impacts are for ages 35–74 years, a trend not mirrored in the least deprived areas.

The results for the cause of death show a similarly contrasting pattern. In the most deprived areas, most causes (15 out of 25) impacted negatively on LE in the second period, in most cases reversing gains recorded in the first period; the difference between the two periods was negative for almost all causes (22 out of 25). In contrast, in the least deprived areas, the difference between the two periods for most causes was marginal. The biggest difference in those areas was for ischaemic heart disease (IHD): improvement fell from 3.9 to 2.0 weeks/year between the two periods. Improvement in IHD also reduced in the most deprived areas; however, in those areas, the biggest negative contribution to LE was from drug-related deaths: this changed from –0.9 weeks/year in the first period to –4.8 weeks/year in the second.

For males (Supplementary Fig. S1), the results were broadly similar. The biggest negative impacts on male LE in the most deprived areas were in the middle-aged groups (35–54 years), and for causes of deaths, the greatest negative differences between the periods were for drug-related deaths, IHD, suicide, and liver cirrhosis.

## Discussion

The analyses confirm that adverse changes in LE in Scotland across the first two decades of the twenty-first century have been most pronounced in the most—rather than least—deprived areas of the country. In those areas, negative impacts on LE have been recorded for the majority of age groups and the majority of causes of death between 2012–14 and 2017–19, reversing improvements observed in the previous decade.

Strengths of the study include the use of mortality data for the whole population residing in the 20% least and most deprived areas in Scotland, and the use of the Arriaga standard decomposition analysis methodology. Limitations include the well-known ecological fallacy, whereby most socioeconomically deprived individuals live outside the 20% most deprived areas [6].

While these analyses do not explore causal relationships between austerity exposures and health outcomes, they do, however, add to—and are consistent with—the existing, extensive, evidence base for the adverse health impacts of UK Government austerity measures in place since 2010 [1]. It has been estimated that between 2010 and 2019, government spending was reduced by more than £500 billion [7], with the greatest cuts made to social security and the funding of local governments, the latter impacting profoundly on the provision of vital public services; those living in the poorest areas of the UK have been disproportionately affected by both components of austerity [1]. Through various poverty- and stress-related causal pathways, including the effects of the loss of income and support services, profound health impacts have been demonstrated across the UK on: child obesity; premature births; a wide range of mental health conditions; multimorbidity; drug-related harm; hospitalizations; and mortality rates and life expectancy [1]. Similar evidence has been demonstrated in other countries where austerity policies were also implemented in the aftermath of the financial crisis in the mid to late 2000s [8].

At the time of writing, the current Labour UK Government (elected in 2024) has only committed to reversing one of several hundred changes made to the UK social security system between 2010 and 2024 [9]; it has also implemented further cuts to those on disability benefits which are likely to cause more harm [10]. It is therefore vital that this government understands the evidence of the harm that these policies cause to the country’s poorest and most vulnerable population.

## Supplementary Material

ckag076_Supplementary_Data

## Data Availability

No new data were generated or analysed in support of this research.

## Rights retention statement

For the purpose of open access, the author(s) have applied a Creative Commons Attribution (CC BY) licence to any Author Accepted Manuscript version arising from this submission.

Key points

- ‘Austerity’ policies in the UK have adversely impacted population health.
- Previous analyses have shown harmful effects on mortality for nearly all age groups and all major causes of death.
- We extend those analyses by stratifying by socioeconomic deprivation, showing that changes between 2012–14 and 2017–19 were substantially worse in poorer areas.
- Politicians need to understand the harmful effects of these policies and adopt different approaches.
