# Supplementary material for: Austerity policies and falling life expectancy in disadvantaged areas in Scotland: a pre-pandemic decomposition analysis
Source: Eur J Public Health. 2026 May 15;36(3):ckag076. doi: 10.1093/eurpub/ckag076 (PMC13178684; doi:10.1093/eurpub/ckag076)
Supplement: ckag076_Supplementary_Data [file ckag076_supplementary_data.docx]

**Supplementary material**

**Table S1. Versions of Scottish Index of Multiple Deprivation (SIMD) used in the analyses**

| **Time period** | **SIMD version** |
| --- | --- |
| 2001-03 | SIMD04 |
| 2004-06 | SIMD06 |
| 2007-09 | SIMD09 |
| 2010-13 | SIMD12 |
| 2014-16 | SIMD16 |
| 2017-19 | SIMD20 |

**Table S2. ICD10 codes**

| Cause of death | ICD10 codes |
| --- | --- |
| Infectious diseases | A00-B99 |
| Lung cancer | C33-C34 |
| Prostate cancer | C61 |
| Breast cancer | C50 |
| Bowel cancer | C18-C21 |
| Other cancers | All other C codes |
| Diabetes | E10-E14 |
| Dementia and Alzheimer's | F01, F03, G30 |
| Mental and behavioural disorders  excluding dementia * | All other F codes |
| Nervous system diseases excluding  Alzheimer’s | All other G codes |
| Ischaemic heart disease | I20-I25 |
| Cerebrovascular | I60-I69 |
| Other circulatory | All other I codes |
| Influenza and pneumonia | J09-J18 |
| Chronic lower respiratory diseases | J40-J47 |
| Other respiratory | All other J codes |
| Digestive excluding cirrhosis | K00-K69  K77-K99 |
| Cirrhosis and other diseases of the liver | K70-K76 |
| Genitourinary | N00-N99 |
| Perinatal conditions | P00-P96 |
| Ill-defined | R00-R99 |
| Suicide and injury/poisoning of  undetermined intent * | X60-X84, Y10-Y34, Y87.0, Y87.2 |
| Accidents * | V01-X59, Y85-Y86 |
| Other external * | All other X&Y codes |
|  | Residual All D; Residual E codes; All H; All L; All O; All M; All Q |
| Drug-related | F11-F15, F19, Plus X40-X44, X60-X64, X85, Y10-Y14 where a controlled drug was present in the body. |

* excluding deaths which are also classified as drug-related

**Table S3. Annualised change in life expectancy (in weeks per year) for populations living in the 20% least and 20% most deprived areas respectively, by sex and time period**

|  | **20% least deprived** | | |  | **20% most deprived** | | |
| --- | --- | --- | --- | --- | --- | --- | --- |
|  | **2001/03 - 2012/14** | **2012/14 - 2017/19** | **Difference** |  | **2001/03 - 2012/14** | **2012/14 - 2017/19** | **Difference** |
| **Males** | 15.2 | 5.8 | -9.4 |  | 17.9 | -8.6 | -26.5 |
| **Females** | 10.4 | 8.4 | -1.9 |  | 8.5 | -8.6 | -17.1 |


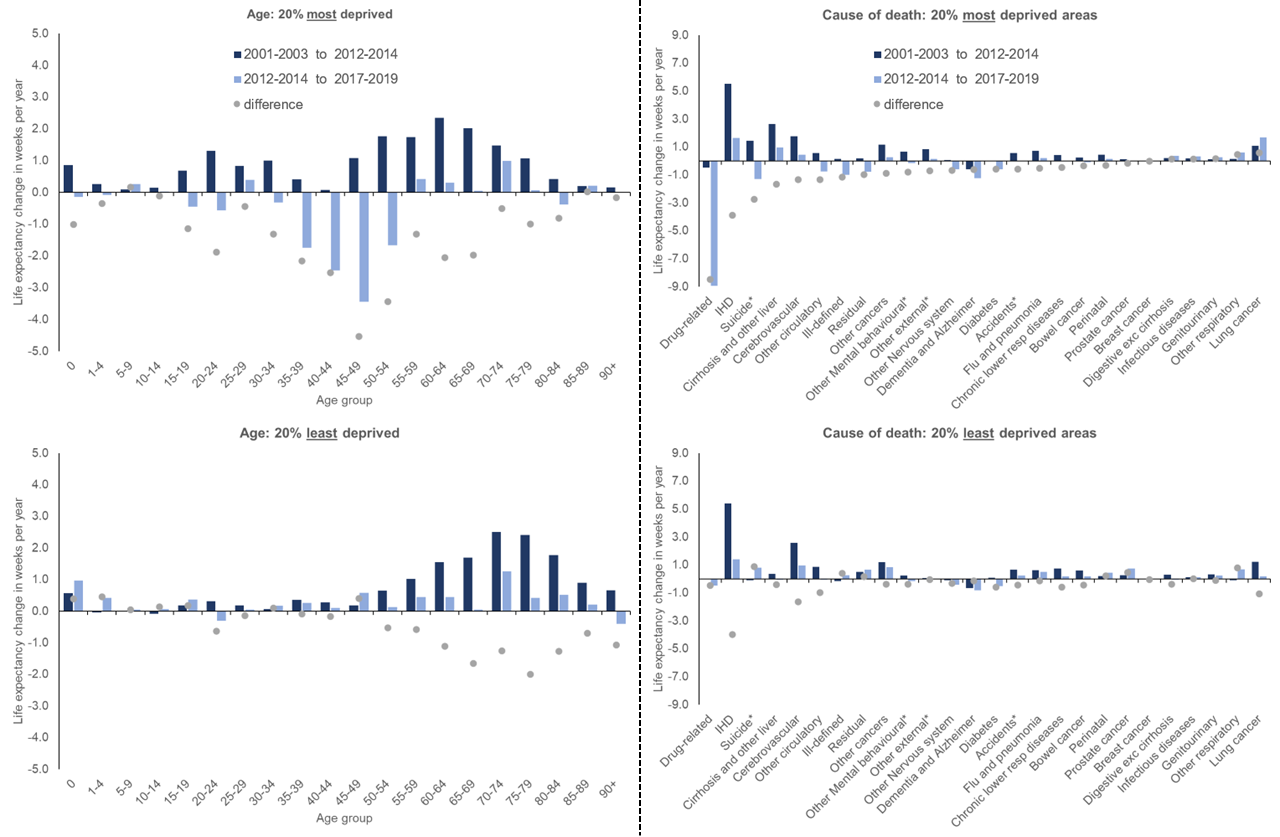


Figure S1. Decomposition of the contribution of age at death (left-hand charts), and specific cause of death (right-hand charts), to changes in male life expectancy between 2001/03 and 2012/14, and 2012/14 and 2017/19, for the populations living in the 20% most deprived (top charts) and 20% least deprived (bottom charts) areas.

** Excluding causes that are included under drug-related deaths. IHD, ischaemic heart disease.*

**Additional references for article**

*As the journal has a maximum number of 10 references for ‘short report’ articles, some had to be excluded from the main manuscript, but are included here.*

**Introduction**:

- Life expectancy declining in poorest areas of UK^^[[1]](#endnote-1)^^[[2]](#endnote-2)^^^^[[3]](#endnote-3)^^^^[[4]](#endnote-4)^^[[5]](#endnote-5)^^[[6]](#endnote-6)^-^^[[7]](#endnote-7)^
- Mortality trends made worse by COVID-19^[[8]](#endnote-8)^…
- …and by ‘cost of living crisis’^[[9]](#endnote-9)^
- Austerity the most likely cause of mortality/life expectancy changes^[[10]](#endnote-10)^^[[11]](#endnote-11)^^^[[12]](#endnote-12)^^^^[[13]](#endnote-13)^^[[14]](#endnote-14)^^^^[[15]](#endnote-15)^^^^[[16]](#endnote-16)^^^^[[17]](#endnote-17)^-^[[18]](#endnote-18)^^

**Methods**

- ‘The break between periods was based on previous estimates of when the LE trends in Scotland changed’^[[19]](#endnote-19)^

**Discussion**

- those living in the poorest areas of the UK have been disproportionately affected by the two main components of austerity^[[20]](#endnote-20)^^[[21]](#endnote-21)^^-^[[22]](#endnote-22)^^
- austerity’s impact on:
  - child obesity^^[[23]](#endnote-23)^^;
  - premature births^^[[24]](#endnote-24)^^;
  - a wide range of mental health conditions^^[[25]](#endnote-25)^^^^[[26]](#endnote-26)^^^^[[27]](#endnote-27)^^^^[[28]](#endnote-28)^^^^[[29]](#endnote-29)^^^^[[30]](#endnote-30)^^[[31]](#endnote-31)^^[[32]](#endnote-32)^^[[33]](#endnote-33)^^[[34]](#endnote-34)^^[[35]](#endnote-35)^^[[36]](#endnote-36)^-^[[37]](#endnote-37)^^;
  - multimorbidity^^[[38]](#endnote-38)^^;
  - drugs harm^^[[39]](#endnote-39)^^ ^^[[40]](#endnote-40)^^;
  - hospitalisations^^[[41]](#endnote-41)^^ ^^[[42]](#endnote-42)^^;
  - mortality rates and life expectancy^13 17 ^[[43]](#endnote-43)^^ ^^[[44]](#endnote-44)^^

*These references:*

1. Fenton L., Wyper G.M., McCartney G., Minton, J. Socioeconomic inequality in recent adverse all-cause mortality trends in Scotland Journal of Epidemiology & Community Health 2019; 73: 971-974 [↑](#endnote-ref-1)
2. Walsh D., McCartney G., Minton J., Parkinson J., Shipton D., Whyte B. Changing mortality trends in countries and cities of the UK: a population-based trend analysis. BMJ Open 2020; 10: e038135 [↑](#endnote-ref-2)
3. Currie J, Boyce T, Evans L, Luker M et al. Life expectancy inequalities in Wales before COVID-19: an exploration of current contributions by age and cause of death and changes between 2002 and 2018. Public Health. 2021; 193: 48-56. [↑](#endnote-ref-3)
4. Rashid T, Bennett JE, Paciorek CJ, et al. Life expectancy and risk of death in 6791 communities in England from 2002 to 2019: high-resolution spatiotemporal analysis of civil registration data. Lancet Public Health 2021; 6(11): e805-e816. [↑](#endnote-ref-4)
5. Walsh D., Wyper G., McCartney G. Trends in healthy life expectancy in the age of austerity. Journal of Epidemiology & Community Health 2022; 76: 743-745. [↑](#endnote-ref-5)
6. Walsh D., Dundas R., McCartney G., Gibson M., Seaman R. Bearing the burden of austerity: how do changing mortality rates in the UK compare between men and women? Journal of Epidemiology & Community Health 2022; 76: 1027-1033 [↑](#endnote-ref-6)
7. de Haro Moro M.T., Schofield L., Munoz-Arroyo R., McCartney G., Walsh D. A new era of inequality: profound changes to mortality in England, Scotland and 10 major British cities. European Journal of Public Health 2025; 35 (2): 235-241 [↑](#endnote-ref-7)
8. Walsh D., McCartney G. Changing mortality rates in Scotland and the UK: an updated summary. Glasgow: Glasgow Centre for Population Health; 2023 [↑](#endnote-ref-8)
9. Richardson E, McCartney G, Taulbut M, Douglas M, Craig N. Population mortality impacts of the rising cost of living in Scotland: scenario modelling study. BMJ Public Health. 2023; 1(1): e000097 [↑](#endnote-ref-9)
10. McCartney G., Walsh D., Fenton L., Devine R. Resetting the course for population health: evidence and recommendations to address stalled mortality improvements in Scotland and the rest of the UK. Glasgow: Glasgow Centre for Population Health 2022 [↑](#endnote-ref-10)
11. Summary of the evidence that austerity has caused the change in mortality trends: Appendix Table A1 (pp 147-8) in: Walsh D., McCartney G. Social murder? Austerity and life expectancy in the UK. Bristol: Bristol Policy Press; 2025 [↑](#endnote-ref-11)
12. Loopstra R., McKee M., Katikireddi S.V., Taylor-Robinson D., Barr B., Stuckler D. Austerity and old-age mortality in England: a longitudinal cross-local area analysis, 2007-2013. Journal of the Royal Society of Medicine 2016; 109(3): 109-16 [↑](#endnote-ref-12)
13. Martin S., Longo F., Lomas J., Claxton K. Causal impact of social care, public health and healthcare expenditure on mortality in England: cross-sectional evidence for 2013/2014. BMJ Open 2021; 11(10): e046417 [↑](#endnote-ref-13)
14. McCartney G, McMaster R, Popham F, Dundas R, Walsh D. Is austerity a cause of slower improvements in mortality in high-income countries? A panel analysis. Social Science & Medicine 2022; 313: 115397 [↑](#endnote-ref-14)
15. Seaman R., Walsh D., Beatty C., McCartney G., Dundas R. Social security cuts and life expectancy: a longitudinal analysis of local authorities in England, Scotland, and Wales. Journal of Epidemiology & Community Health 2024; 78: 82-87. [↑](#endnote-ref-15)
16. Watkins J, Wulaningsih W, Da Zhou C, Marshall DC, Sylianteng GDC, Dela Rosa PG, Miguel VA, Raine R, King LP, Maruthappu M. Effects of health and social care spending constraints on mortality in England: a time trend analysis. BMJ Open 2017; 7(11): e017722. [↑](#endnote-ref-16)
17. Alexiou A, Fahy K, Mason K. et al. Local government funding and life expectancy in England: a longitudinal ecological study. Lancet Public Health 2021; 6(9): e641-e647. [↑](#endnote-ref-17)
18. Price T. “They pulled that funding away and we’re not recovering. It’s getting worse”: deaths of despair in post-austerity north east England. Int J Equity Health 2024; 23: 242. [↑](#endnote-ref-18)
19. Fenton L., Minton J., Ramsay J., Kaye-Bardgett M., Fischbacher C., Wyper G.M.A., McCartney G. Recent adverse mortality trends in Scotland: comparison with other high-income countries. BMJ Open. 2019; 9(10): e029936 [↑](#endnote-ref-19)
20. Gray M., Barford A. The depths of the cuts: The uneven geography of local government austerity. Cambridge Journal of Regions, Economy and Society 2018; 11 (3): 541-563 [↑](#endnote-ref-20)
21. Beatty C., Fothergill S. The uneven impact of welfare reform: the financial losses to places and people. Sheffield: Sheffield Hallam University; 2016. [↑](#endnote-ref-21)
22. Beatty C., Fothergill S. Hitting the poorest places hardest: the local and regional impact of welfare reform. Sheffield: Sheffield Hallam University; 2013 [↑](#endnote-ref-22)
23. Mason KE, Alexiou A, Bennett DL, et al Impact of cuts to local government spending on Sure Start children’s centres on childhood obesity in England: a longitudinal ecological study J Epidemiol Community Health 2021; 75: 860-866. [↑](#endnote-ref-23)
24. Watson R., Walsh D., Scott S., Carruthers J., Fenton L., McCartney G., Moore E. Is the period of austerity in the UK associated with increased rates of adverse birth outcomes? European Journal of Public Health 2024; 34 (6): 1043-1051 [↑](#endnote-ref-24)
25. Reeves A., Clair A., McKee M., Stuckler D. Reductions in the United Kingdom's Government Housing Benefit and Symptoms of Depression in Low-Income Households. Am J Epidemiol. 2016; 184(6): 421-9. [↑](#endnote-ref-25)
26. Katikireddi SV, Molaodi OR, Gibson M, Dundas R, Craig P. Effects of restrictions to Income Support on health of lone mothers in the UK: a natural experiment study. Lancet Public Health. 2018 Jul;3(7): e333-e340 [↑](#endnote-ref-26)
27. Cherrie M, Curtis S, Baranyi G, Cunningham N, Dibben C, Bambra C, Pearce J. A data linkage study of the effects of the Great Recession and austerity on antidepressant prescription usage. Eur J Public Health 2021; 31(2): 297-303. [↑](#endnote-ref-27)
28. Wickham S, Bentley L, Rose T, Whitehead M, Taylor-Robinson D, Barr B. Effects on mental health of a UK welfare reform, Universal Credit: a longitudinal controlled study. Lancet Public Health. 2020; 5(3): e157-e164. [↑](#endnote-ref-28)
29. Thomson RM, Niedzwiedz CL, Katikireddi SV. Trends in gender and socioeconomic inequalities in mental health following the Great Recession and subsequent austerity policies: a repeat cross-sectional analysis of the Health Surveys for England. BMJ Open 2018; 8(8): e022924. [↑](#endnote-ref-29)
30. Reeves, A., Fransham, M., Stewart, K., Patrick, R. Does capping social security harm health? A natural experiment in the UK. Social Policy & Administration 2022; 56 (3): 345-359. [↑](#endnote-ref-30)
31. Kim C., Teo C., Nielsen A., Chum A. What are the mental health consequences of austerity measures in public housing? A quasi-experimental study. J Epidemiol Community Health 2022; 76:730–735 [↑](#endnote-ref-31)
32. Barr B, Taylor-Robinson D, Stuckler D, Loopstra R, Reeves A, Whitehead M. 'First, do no harm': are disability assessments associated with adverse trends in mental health? A longitudinal ecological study. J Epidemiol Community Health 2016; 70(4): 339-45. [↑](#endnote-ref-32)
33. Barr B., Kinderman P., Whitehead M. Trends in mental health inequalities in England during a period of recession, austerity and welfare reform 2004 to 2013. Soc Sci Med. 2015; 147: 324-31; [↑](#endnote-ref-33)
34. Brown H., Gao N., Song W. Regional trends in mental health inequalities in young people aged 16-25 in the UK and the role of cuts to local government expenditure: Repeated cross-sectional analysis using the British household panel Survey/UK household longitudinal survey. Soc Sci Med. 2024; 353: 117068. [↑](#endnote-ref-34)
35. Thornton I, Iacoella F. Conditionality and contentment: Universal Credit and UK welfare benefit recipients’ life satisfaction. Journal of Social Policy 2024; 53(2): 280-308. [↑](#endnote-ref-35)
36. Gascoigne C., Jeffery A., Shao Z., Geneletti S. et al. A Bayesian Interrupted Time Series framework for evaluating policy change on mental well-being: An application to England's welfare reform. Spat Spatiotemporal Epidemiol. 2024; 50:100662 [↑](#endnote-ref-36)
37. Fahy, K., Alexiou, A., Daras, K. et al. Mental health impact of cuts to local government spending on cultural, environmental and planning services in England: a longitudinal ecological study. BMC Public Health 2023; 23: 1441 [↑](#endnote-ref-37)
38. Stokes J., Bower P., Guthrie B., Mercer S.W., Rice N., Ryan A.M., Sutton M. Cuts to local government spending, multimorbidity and health-related quality of life: A longitudinal ecological study in England. The Lancet Regional Health – Europe 2022; 100436. [↑](#endnote-ref-38)
39. Alexiou A., Mason K., Fahy K. et al. Assessing the impact of funding cuts to local housing services on drug and alcohol related mortality: a longitudinal study using area-level data in England. International Journal of Housing Policy 2021. [↑](#endnote-ref-39)
40. Koltai J., McKee M., Stuckler D. Association between disability-related budget reductions and increasing drug-related mortality across local authorities in Great Britain. Social Science & Medicine 2021; 284: 114225 [↑](#endnote-ref-40)
41. Friebel R, Yoo KJ, Maynou L. Opioid abuse and austerity: Evidence on health service use and mortality in England. Soc Sci Med. 2021: 114511. [↑](#endnote-ref-41)
42. Crawford R., Stoye G., Zaranko B. Long-term care spending and hospital use among the older population in England. J Health Econ. 2021; 78: 102477. [↑](#endnote-ref-42)
43. Richardson E., Taulbut M., Robinson M., Pulford A., McCartney G. The contribution of changes to tax and social security to stalled life expectancy trends in Scotland: a modelling study. Journal of Epidemiology & Community Health 2020; 75(4): 365–70 [↑](#endnote-ref-43)
44. Berman Y., Hovland T. The Impact of Austerity on Mortality and Life Expectancy. London: London School of Economics (LSE); 2024 [↑](#endnote-ref-44)
